# Association of cystatin C proteoforms with estimated glomerular filtration rate

**DOI:** 10.1016/j.clinms.2016.11.001

**Published:** 2016-11-14

**Authors:** Olgica Trenchevska, Juraj Koska, Shripad Sinari, Hussein Yassine, Peter D. Reaven, Dean D. Billheimer, Randall W. Nelson, Dobrin Nedelkov

**Affiliations:** a The Biodesign Institute, Arizona State University, Tempe, AZ, USA; b Department of Medicine, Phoenix Veteran Affairs Medical Center, Phoenix, AZ, USA; c Biostatics Consulting Lab, University of Arizona, Tucson, AZ, USA; d Department of Medicine, University of Southern California, Los Angeles, USA

**Keywords:** Cystatin C, Proteoforms, GFR, Mass spectrometry, Immunoassay

## Abstract

**Background:**

Cystatin C (CysC), a marker for chronic kidney disease, exists as three sequence proteoforms, in addition to the wild-type sequence: one contains hydroxyproline at position 3 (3Pro-OH), the two others have truncated sequences (des-S and des-SSP). Here, we examine correlations between each of these CysC proteoforms and estimated glomerular filtration rate (eGFR), a diagnostic criterion for chronic kidney disease (CKD).

**Methods:**

CysC proteoform concentrations were determined from the plasma of 297 diabetes patients at a baseline time point and nine-months later, using a mass spectrometric immunoassay, and were correlated with eGFR calculations.

**Results:**

In all samples, 3Pro-OH was the most abundant CysC proteoform, followed by the wild-type proteoform. Least abundant were the truncated CysC proteoforms, des-S and des-SSP, although they demonstrated stronger negative correlation with eGFR than the 3Pro-OH and wild-type proteoforms. The des-SSP truncated proteoform exhibited negative predictive value for eGFR.

**Conclusions:**

The truncated CysC proteoforms show potential for clinical and prognostic utility in CKD staging. This could be useful in populations where current methods do not provide satisfactory solutions.

## Highlights

• Cystatin C, a marker of chronic kidney disease (CKD), exists as multiple proteoforms.
• Mass spectrometry can discriminate between the individual cystatin C proteoforms.
• We examined the association between cystatin C proteoforms and estimated glomerular filtration rate.
• Truncated cystatin C proteoforms showed strong negative association with eGFR.
• The des-SSP truncated proteoform exhibited negative predictive value for eGFR.

## 1 Introduction

Cystatin C (CysC) is a cysteine proteinase inhibitor encoded by the CST3 gene, a member of the type 2 cystatin gene family [1]. Following cleavage of an N-terminal signal peptide, the mature form of CysC consists of a 120 amino acid polypeptide chain, with two disulfide bridges and a molecular mass of 13,343 Da. CysC exists *in vivo* as several posttranslationally-modified variants, or “proteoforms” [2]: the first proteoform has hydroxyproline at position 3 (3Pro-OH), while the other two variants have truncated sequences – one missing the N-terminal serine (des-S), and the other missing the first three N-terminal residues (des-SSP). Our group has detected these proteoforms in human plasma [3], [4] and urine [5] using a mass spectrometric immunoassay (MSIA) method that combines protein affinity retrieval using antibodies and MALDI-TOF for proteoform identification [6], [7]. This method differs from standard ELISAs in that it uses mass spectrometry (MS) to provide direct readout of the intact molecular mass – an intrinsic property of each proteoform. In contrast, the secondary (i.e., labeled) reporter antibody in ELISAs cannot discriminate between structural protein modifications; hence, the resulting quantitative signal is a summation of signals from all proteoforms for a given protein that are captured by the primary antibody.

Recently, we have developed and validated a quantitative version of the MSIA for CysC that uses beta-lactoglobulin (BL) as an internal reference standard [8]. BL was chosen, because it is not a human protein, and has a molecular mass of 18,281 Da, which is near that of human CysC. Quantitation was achieved by affinity co-purifying BL along with CysC using antibody-derivatized affinity pipettes, normalizing the CysC proteoform values based on BL recovery, and, determining protein concentration from the normalized signal intensity based on a standard curve. We have applied this quantitative MSIA to a cohort of 500 healthy individuals to determine the concentration ranges of these three proteoforms and the wild-type CysC in the healthy population [9].

It was determined that 3Pro-OH was the most abundant CysC proteoform, followed by the wild-type protein; least abundant were the two truncated CysC proteoforms, des-S and des-SSP. The existence and percentages of these proteoforms in human sera were recently confirmed independently by another research group using immuno-MALDI-MS [10]. The CysC proteoforms have also been detected in fresh human plasma samples [9], indicating they are most likely created *in vivo*, through protein/enzymatic processing [11]. While it has been shown that leucocyte elastase cleaves the Val10-Gly11 bond of CysC [12], and cathepsin L cleaves the Gly11-Gly12 bond [13], the exact mechanism for the creation of the CysC truncated proteoforms analyzed here remains unknown. The N-terminus of CysC is unstructured and highly mobile [14], and it contains the inhibitory binding site for the cysteine proteinases. Removal of the first ten amino acids drastically reduces CysC inhibition of several cysteine proteinases as a result of decreased affinity [12], [15]. Hence, the truncated des-S and des-SSP CysC proteoforms may also exhibit reduced biological activity.

CysC is an emerging biomarker for the diagnosis and monitoring of chronic kidney disease (CKD) [16]. CKD is defined by a persistent reduction in the glomerular filtration rate (GFR) to below 60 mL/min per 1.73 m^2^ for three or more months [17]. The level of GFR and the magnitude of its change over time are important for CKD detection and severity assessment. The urinary or plasma clearance of an ideal filtration marker, such as inulin, is the gold standard for the measurement of GFR [18]. This method is cumbersome, however, leading investigators to use serum levels of endogenous filtration markers, such as creatinine, to obtain an estimated GFR (eGFR) instead.

Two commonly used equations to calculate eGFR are the Modification of Diet in Renal Disease (MDRD) study equation and the Cockcroft-Gault equation [19], [20]. Both equations use serum creatinine in combination with age, sex, weight or race to estimate GFR. Another equation that also uses serum creatinine is the Chronic Disease Epidemiology Collaboration (CKD-EPI) [21]. However, all of these equations have shortcomings. The MDRD Study equation works best in the lower ranges of GFR [22]. GFR estimates using the MDRD Study equation that are greater than 60 mL/min per 1.73 m^2^ underestimate measured GFR and may lead to misdiagnosis or misclassification of CKD in individuals with mild renal insufficiency [23], [24]. Additionally, all equations underestimate GFR in the elderly, especially at higher GFRs [25], [26].

Because CysC levels are less influenced by muscle mass and diet than creatinine, CysC is being considered as a novel endogenous filtration marker for the estimation of GFR [27]. Some studies have reported GFR-estimating equations based on serum levels of CysC, either alone or in combination with serum creatinine [28]; however, those equations are not accurate for all populations [29]. CysC has been indicated as an improved predictor of adverse effects in the elderly, including mortality, heart failure, bone loss, peripheral arterial disease and cognitive impairment [30], [31].

Because of the increased use of CysC in eGFR equations, we investigated the correlation of individual CysC proteoforms with eGFR. We have recently correlated proteoforms of other proteins with diseases and outcomes in a number of clinical cohorts [32], [33], [34], [35], [36]. In the current work, we apply the quantitative CysC proteoforms assay to a cohort of diabetes patients to examine the relationship between CysC proteoforms and eGFR.

## 2 Materials and methods

### 2.1 Study population

Human plasma samples were obtained from the Risk Factors, Atherosclerosis and Clinical Events in Diabetes (RACED) cohort, a seven-center sub-study of the Veterans Affairs Diabetes Trial [37]. Patients were all carefully assessed by medical history, physical exam and blood work at the time of study enrollment and only those without active problems needing diabetes treatment were enrolled. Samples from 297 patients with type 2 diabetes were available at a baseline time point (Visit 1, the beginning of the study) and at 9 months (Visit 2); this was a glucose lowering trial, and the HbA1c values decreased and stabilized by 9 months into the study. The current study and use of human samples were approved by Arizona State University’s Institutional Review Board #1106006545.

### 2.2 Mass spectrometric immunoassay (MSIA)

CysC proteoform concentrations were determined by MSIA, as previously described [8]. Briefly, antibodies against CysC (Cat. No. GCYS-80A, ICLLab, Portland, OR, USA) and the internal reference standard, BL (Cat. No. GTX77272, Genetex, Irvine, CA, USA) were co-immobilized onto microcolumns at the entrance of pipette tips, in an empirically-determined optimal mass ratio of 4.5:1 (CysC:BL). For the standard curves, commercially available CysC (Cat. No. CRC173B, Cell Sciences, Canton, MA, USA) was serially diluted in PBS buffer containing 3 g/L BSA to final concentrations of 1.25, 0.625, 0.313, 0.156, 0.078, and 0.039 mg/L. The human plasma samples were diluted 5-fold in PBS, 0.1% Tween. Analytical samples were prepared by combining 20 μL of the CysC standards or diluted plasma with 40 μL of 1 mg/mL BL and 100 μL PBS 0.1% Tween, into the wells of a microtiter plate.

The antibody-derivatized pipette tips were mounted on the head of an automated multichannel pipettor (Multimek 96, Beckman Coulter, Brea, CA, USA), immersed into microtiter plate wells containing 160 μL of the analytical samples, and 250 aspiration and dispense cycles were performed (100 μL volumes each) allowing for affinity capture of the proteins (room temperature, ∼10 min). Following rinses with PBS 0.1% Tween buffer (100 cycles, 100 μL volumes) and water (2 × 10 cycles, 100 μL volumes, ∼5 min), the affinity pipettes were immersed into microplate wells containing MALDI matrix solution (25 g/L sinapic acid in aqueous solution containing 33% (v/v) acetonitrile, and 0.4% (v/v) trifluoroacetic acid), and 6 μL were aspirated into the affinity pipettes. After a 10 s delay, to allow for the dissociation of the protein from the capturing antibody, the matrix containing the proteins was dispensed directly onto a 96-well formatted MALDI target. Linear mass spectra were acquired on a Bruker *Autoflex* III MALDI-TOF mass spectrometer, with a delayed extraction mode using a 1.4 kV draw out pulse, 150 ns delay, and a full accelerating potential of 20 kV. In order to obtain accurate mass spectra representation, 10,000 laser shots were collected from various areas of each sample and summed into a single mass spectrum. The mass spectra were processed (baseline subtracted and smoothed) using Flex Analysis software (Bruker Daltonics).

Protein signals in the mass spectra were assigned based on accurately measured *m*/*z* values and our previous work, which included tryptic digestion of the eluted proteins to confirm the identities of the CysC proteoforms [8]. Peak heights for the CysC and BL signals were measured, and the ratios of the CysC/BL peak heights were calculated for the CysC standards samples. Standard curves were generated by plotting the CysC/BL ratios against the concentration of the human CysC standards. The CysC/BL peak heights ratios for each CysC proteoform in the plasma samples were then calculated, and the sum of the ratios was used to determine the total cystatin C concentration using the standard curves. The concentrations of the individual proteoforms were calculated based on their percentage representation of total CysC concentration.

### 2.3 Analysis of population data

eGFR at both Visits 1 and 2 was calculated from creatinine measurements as described [37], using the Cockcroft-Gault equation. For the Spearman correlations with eGFR, the CysC proteoform concentrations, determined from the MSIA, were used, in addition to their log ratios with respect to the wild-type CysC proteoform. An analysis of covariance (ANCOVA) for the eGFR values at Visit 2 was performed, with baseline values of the CysC proteoforms (concentrations and log ratios to wild-type CysC), using values for eGFR at Visit 1 as covariates.

## 3 Results and discussion

**Fig. 1:**
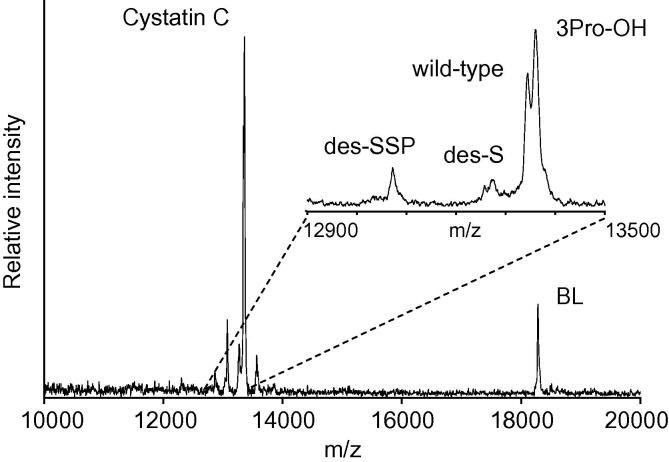
Representative mass spectrum obtained with the CysC MSIA. CysC signals are observed form the wild-type, 3Pro-OH, des-S, and des-SSP proteoforms. BL – beta lactoglobulin, served as an internal reference standard.

A representative mass spectrum resulting from the analysis of the human plasma samples is shown in Fig. 1. Present in the spectrum are only signals for the two targeted proteins, CysC and BL (i.e. internal reference standard), without any other signals resulting from non-specifically bound proteins, demonstrating the specificity of the assay and the selectivity of the antibodies. The inset spectrum in Fig. 1 shows the wild-type CysC protein signal, along with the three proteoforms: 3Pro-OH, des-S, and des-SSP. These proteoforms were observed in every sample from the cohort.

**Fig. 2:**
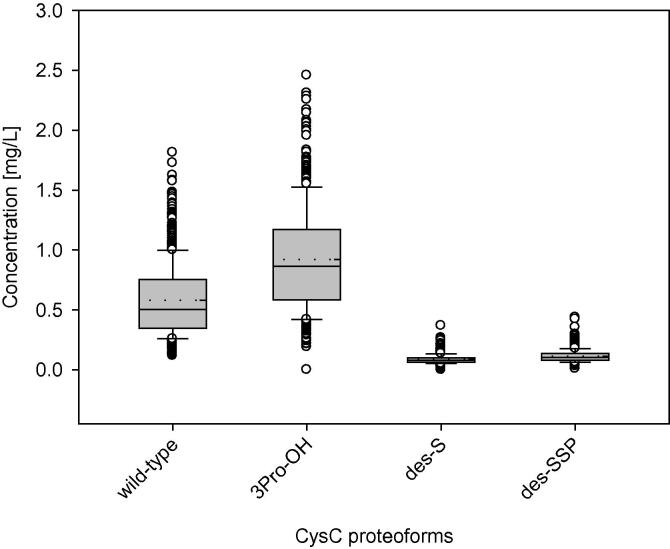
Individual concentrations of cystatin C proteoforms. Box – 25–75th percentile; Solid line – median concentration; Short dash line – mean concentration; Error bars – 10th and 90th percentile; Symbols – outlying points.

The concentrations of the individual CysC proteoforms were determined using the quantitative MALDI-TOF MSIA and the standard curves. While some doubts have been raised regarding the quantitative nature of MALDI MS, our group [38], [39], [40], [41], [42] and others [43], [44], [45], [46], [47] have successfully developed MALDI-based protein quantification assays with excellent CVs and reproducibility. Fundamental to accurate quantitative analysis using MALDI are proper matrix preparation and application, as well as reduction of shot-to-shot variation, which was achieve through summation of a large number of laser shots across each sample spot into a single mass spectrum. With these parameters optimized, peak areas or peak heights can be used for protein quantification. In this work, peak heights were used instead of peak areas to allow for the quantification of the wild-type protein and 3Pro-OH signals that were not baseline resolved in the mass spectra (Fig. 1 inset). Others have found peak heights to be more accurate than peak areas for CysC MALDI-TOF MS quantification [10], as well as for other analytes [48], [49]. As previously determined, the assay exhibits good intra- and inter-assay precision (<10% CVs), linearity and spiking-recovery in the range of 90–110%, and a slight positive bias when compared to an IVD-approved ELISA test, which may be attributed to the possibility that the ELISA may not measure all of the proteoforms [8]. The concentrations of the CysC proteoforms in the entire cohort are shown in Fig. 2. Data from both visits were plotted together, as there was no statistically significant difference among the values for the two visits (results not shown). As observed previously in a healthy cohort of regular blood donors [9], 3Pro-OH is the most abundant proteoform; the wild-type protein was a smaller fraction of the total CysC. The 3Pro-OH to wild-type CysC ratio was ∼1.25:1, which is consistent with previous studies that utilized amino acid sequencing/HPLC [50], and papain affinity chromatography/MALDI-TOF MS [51]. The total CysC concentration observed in this diabetes cohort (mean = 1.70 mg/L) was higher than that obtained for healthy individuals [9] (mean = 1.03 mg/L). Similar trends were observed in another study of CysC concentrations in type 2 diabetes patients [52].

Spearman correlation analyses were performed to examine the relationship between the concentrations of the CysC proteoforms and eGFR, which was estimated from serum creatinine measurements. The data are shown in Table 1. The two truncated CysC proteoforms (des-S and des-SSP) show stronger negative correlation with eGFR than the 3Pro-OH and wild-type proteoforms. When log ratios of the three proteoforms, with respect to wild-type CysC, are used (normalizing to the wild-type protein signal), the correlations become weaker; however, the negative association between des-SSP and eGFR remains strong. Notably, correlations were not detected in this study between the CysC proteoform concentrations and other clinical measures and characteristics of the cohort [53].

**Table 1:** Spearman correlations for cystatin C proteoforms (concentrations, and log ratios to wild-type cystatin C) with eGFR, at baseline (Visit 1) and after nine months (Visit 2).

	eGFR	
	Visit 1	Visit 2
Concentrations		
des-SSP	−0.43 (p<0.001)	−0.38 (p < 0.001)
des-S	−0.37 (p < 0.001)	−0.37 (p < 0.001)
3Pro-OH	−0.21 (p < 0.001)	−0.15 (p = 0.02)
wild-type	−0.21 (p < 0.001)	−0.13 (p = 0.05)

*Log ratio to wild-type*		
des-SSP	−0.33 (p < 0.001)	−0.38 (p < 0.001)
des-S	−0.19 (p < 0.001)	−0.22 (p < 0.001)
3Pro-OH	−0.03 (p = 0.6)	−0.06 (p = 0.3)

Similar results were observed in another recently published study [54]. Using the same quantitative MSIA on a cohort of diabetic CKD patients and controls, it was determined that the CysC proteoforms had higher concentrations in the diabetic CKD group than the healthy control group. Similar to what was observed in the current study, the negative association with eGFR was stronger for the truncated CysC proteoforms than wild-type. These results suggest that the truncated proteoforms may not be cleared through the renal glomeruli as quickly as wild-type CysC in CKD.

Because the cohort used in the current study consisted of samples obtained from two visits, nine months apart, we further examined the role of the CysC proteoforms as eGFR predictors. We analyzed the eGFR values at Visit 2 as a function of the baseline values of the CysC proteoforms, adjusted for the initial eGFR (Visit 1, baseline). The only proteoform that showed a negative association with eGFR was the des-SSP truncated form. The strength of the association was maintained throughout the concentration range (slope coefficient of −0.06) and the log ratio to wild-type values (slope coefficient of −0.08), all with p < 0.05. These data suggest a negative relationship between des-SSP levels and future GFR values, indicating a possible role of this proteoform as a predictor of GFR changes and CKD progression.

## 4 Conclusion

Proteoforms could have undiscovered pathophysiological implications and potential clinical utility for specific diseases. With the capability to detect and quantify a number of CysC proteoforms, we are able to explore their relationship with specific clinical metrics and outcomes. In this work, we examined the relationship between CysC proteoforms and eGFR in a cohort of diabetic patients, identifying a strong negative association for truncated proteoforms and predictive power for changes in GFR for the most-truncated proteoform. The truncated CysC proteoforms could have clinical and prognostic significance in CKD staging, especially in populations where the current equations do not provide satisfactory solutions. Studies with larger longitudinal cohorts are warranted to further explore these associations. If, and when, such associations are identified and validated, the CysC MSIA could readily be adapted for clinical use [55].

## Author contributions

All the authors have accepted responsibility for the entire content of this submitted manuscript and approved submission.

## Research funding

OT, JK, SS, HY, PDR, DDB, RWN, and DN were supported by Grants R01DK082542 and R24DK090958 from the NIDDK. JK and PDR were supported by the Cooperative Studies Program of the Department of Veterans Affairs Office of Research and Development, and by 10.13039/100000050NHLBI Grant R01HL067690. SS and DDB were supported by 10.13039/100000066NIEHS Grant P30 ES006694. HY was supported by Grant K23HL107389 from the NHLBI, 12CRP11750017 from the American Heart Association, and USC CTSI pilot UL1TR000130.

## Employment or leadership

None declared.

## Honorarium

None declared.

## Competing interests

The funding organization(s) played no role in the study design; in the collection, analysis, and interpretation of data; in the writing of the report; or in the decision to submit the report for publication.
